# Extraintestinal Cancers in Inflammatory Bowel Disease: A Literature Review

**DOI:** 10.3390/cancers15153824

**Published:** 2023-07-27

**Authors:** Alessandro Massano, Luisa Bertin, Fabiana Zingone, Andrea Buda, Pierfrancesco Visaggi, Lorenzo Bertani, Nicola de Bortoli, Matteo Fassan, Marco Scarpa, Cesare Ruffolo, Imerio Angriman, Cristina Bezzio, Valentina Casini, Davide Giuseppe Ribaldone, Edoardo Vincenzo Savarino, Brigida Barberio

**Affiliations:** 1 Gastroenterology Unit, Department of Surgery, Oncology and Gastroenterology, University Hospital of Padova, 35128 Padova, Italy; alessandro.massano@studenti.unipd.it (A.M.); luisa.bertin@studenti.unipd.it (L.B.); fabiana.zingone@unipd.it (F.Z.); brigida.barberio@unipd.it (B.B.); 2 Gastroenterology Unit, Department of Gastrointestinal Oncological Surgery, S. Maria del Prato Hospital, 32032 Feltre, Italy; andrea.buda66@gmail.com; 3 Gastroenterology Unit, Department of Translational Research and New Technologies in Medicine and Surgery, University of Pisa, 56126 Pisa, Italy; pierfrancesco.visaggi@gmail.com (P.V.); lorenzobertani@gmail.com (L.B.); nicola.debortoli@unipi.it (N.d.B.); 4 Surgical Pathology Unit, Department of Medicine, University of Padova, 35138 Padova, Italy; matteo.fassan@unipd.it; 5 General Surgery Unit, Department of Surgery, Oncology and Gastroenterology, University of Padova, 35138 Padova, Italy; marcoscarpa73@yahoo.it (M.S.); cesare.ruffolo@aopd.veneto.it (C.R.); imerio.angriman@unipd.it (I.A.); 6 IBD Center, Gastroenterology Unit, Rho Hospital, ASST Rhodense, 20017 Rho, Italy; cribezzio03@yahoo.it; 7 Gastroenterology Unit, ASST Bergamo Est, 24068 Seriate, Italy; valentina.casini@gmail.com; 8 Department of Medical Sciences, Division of Gastroenterology, University of Turin, 10126 Turin, Italy; davidegiuseppe.ribaldone@unito.it

**Keywords:** inflammatory bowel disease, malignancy, extraintestinal cancers, biologic agents

## Abstract

**Simple Summary:**

Crohn’s disease (CD) and ulcerative colitis (UC) are chronic inflammatory bowel diseases that affect the gut and extraintestinal organs. Preliminary evidence has shown that patients with CD or UC are at increased risk of developing intestinal and extraintestinal cancers, therefore, there is an ever-growing concern about the safety of immunomodulators and biologics for these patients. The aim of this review is to summarize the evidence regarding the association between Inflammatory Bowel Disease (IBD) and extraintestinal cancers, and the safety and management of immunomodulators and biologics for patients with IBD and previous or current extraintestinal cancer.

**Abstract:**

Background: Inflammatory bowel disease (IBD) is a group of chronic multifactorial inflammatory disorders including two major entities: Crohn’s disease (CD) and ulcerative colitis (UC). Preliminary evidence suggests that patients with IBD may be at increased risk of developing intestinal and extraintestinal cancers (EICs). Actually, little is known about the association between IBD and EICs, and there is ever-growing concern regarding the safety of immunomodulators and biological therapy, which may represent a risk factor for carcinogenesis. Aims: The aim of this review is to summarize the evidence regarding the association between IBD and EICs, the safety of immunomodulators and biological therapy and the management of immunomodulators and biologic agents in IBD patients with prior or current EICs. Results: IBD patients have a higher risk of developing different forms of extraintestinal solid organ tumors and hematological malignancies. Immunomodulators and biological therapy may increase the risk of developing some types of EICs and may be consciously used in patients with IBD and current or prior history of malignancy. Conclusions: Decisions regarding the use of immunomodulators or biological therapies should be made on an individual basis, considering a multidisciplinary approach involving oncologists.

## 1. Introduction

Inflammatory bowel disease (IBD), including Crohn’s disease (CD) and ulcerative colitis (UC), is a group of chronic, multifactorial disorders affecting the gastrointestinal tract. The etiopathogenesis of IBD is still uncertain, but actual knowledge of the disease suggests that its onset originates from genetic and environmental factors, alongside impaired intestinal permeability, gut dysbiosis, autoinflammatory pathways, and immunological dysregulation [1]. Nearly 7 million people suffer from IBD worldwide, mostly in Europe (2 million) and America (1.5 million) and the incidence of the disease is rising [2]. Both CD and UC lead to chronic inflammation of the gut, but inflammation may also affect other organs, causing extraintestinal manifestations [3,4,5,6]. Moreover, both conditions are associated with impaired quality of life and increased disability [7,8,9].

Chronic inflammatory insults of the gut may increase the risk of cancer formation in patients with IBD, such as colorectal cancer [7,8,9]. Risk factors for the development of intestinal cancers are well known, including clinical characteristics of the patients or phenotypes of the disease, and there are precise indications for screening, surveillance, and management of these patients [7]. Actually, several studies have demonstrated that the incidence of intestinal cancer is decreasing in IBD patients, due to greater control of intestinal inflammation—likely secondary to better knowledge of the disease and more readily available drugs than in the past—and to the improvement in endoscopic surveillance and resection strategies [10].

On the other hand, less is known about the association between IBD and EICs [7]. Overall, the global prevalence of cancers is increasing in the general population, and also in patients with IBD [11]. Therefore, gastroenterologists are increasingly caring for patients with IBD and current cancer or a previous history of cancer, being faced with making therapeutic decisions on an individual basis [11,12]. 

Current treatment of IBD includes immunomodulators (such as thiopurines and methotrexate) and biological drugs, that may act on the immune system and promote carcinogenesis, leading to the formation or recurrence of malignancies [13]. Therefore, there is an ever-growing concern about these drugs for their supposed carcinogenic effect, so they are usually discontinued in patients with IBD [13]. However, more evidence is needed to support these assumptions.

Several studies have supported that patients with IBD do not have an increased overall risk of EICs; however, it seems that they have a higher risk of specific EICs than healthy controls [14,15,16]. 

The aim of this review is to summarize the current evidence regarding:

1. The association between IBD and EICs.
2. The safety of immunomodulators and biological drugs, considering their possible association with the risk of developing EICs.
3. Therapy management with immunomodulators and biologic agents in patients with IBD and prior or current EICs.

## 2. Materials and Methods

A literature search from the bibliographic database of PubMed was performed using the following keywords: “Inflammatory Bowel Disease”, “IBD”, “Crohn’s disease”, “ulcerative colitis”, “extraintestinal cancer”, “extraintestinal malignancies”, “thiopurines”, “immunomodulators”, “biological therapy”, “TNF”, “vedolizumab”, “ustekinumab”, and “tofacitinib”. Overall, 562 papers written in English and published from March 1975 to April 2023 were identified. Titles and abstracts of the papers were evaluated by two authors (AM and LB). Most relevant articles related to extraintestinal malignancies in IBD were selected and read in full.

Articles were then divided into three sections:

1. Articles that estimated the association between IBD and EICs.
2. Articles that evaluated the safety of immunomodulators and biological drugs.
3. Articles that evaluated the therapy management with immunomodulators and biological drugs in patients with IBD and prior or current diagnosis of EICs.

## 3. Results

### 3.1. Association between IBD and EICs

#### 3.1.1. Solid-Organ Tumor

We included 32 articles that analyzed the association between IBD and solid-organ tumors: the main findings of each study are summarized in Table 1 [17,18,19,20,21,22,23,24,25,26,27,28,29,30,31,32,33,34,35,36,37,38,39,40,41,42,43,44,45,46,47,48].

Several studies did not find an overall risk of solid-organ tumors in patients with IBD when compared with a healthy population [17,18,19,20,21,30,34,39]. However, this might have changed when patients were stratified based on disease type or disease location. In fact, Ekbom et al. found an increased risk of solid EICs in patients with ulcerative proctitis (standardized incidence ratio, SIR = 1.3; 95% CI, 1.0–1.7) [17]; while Jess et al. and Bernstein et al. demonstrated that the risk was higher in patients with CD (SIR = 1.55; 95% CI, 1.29–1.84; and incidence rate ratio, IRR = 1.29; 95% CI, 1.07–1.54, respectively) [20,30]. Moreover, some evidence has shown that certain comorbidities such as chronic kidney disease, respiratory disease, and diabetes mellitus may be associated with carcinogenesis in patients with IBD (odds ratio, OR = 1.29, 1.07, and 1.06, respectively) [42].

Although a higher overall risk of solid EICs has not been demonstrated, studies have shown an increased risk in patients with IBD of some specific solid tumors including biliary cancers, liver cancer, pancreatic cancer, non-melanoma skin cancers (NMSC), reproductive cancers, urological malignancies, respiratory malignancies, and thyroid cancer [17,18,19,20,22,23,24,26,28,29,30,31,32,37,38,39,40,41,42,43,44,46,47,48,49]. 

Particularly, several studies and meta-analyses have shown an increased risk of biliary cancers in patients with IBD, mainly cholangiocarcinoma (CCA) (risk ratio, RR = 2.63; 95% CI, 1.47–4.72), and particularly in subjects with UC (RR = 3.40; 95% CI, 2.50–4.62) [19,20,26,29,31,36,38,46,49]. Intrahepatic CCA (iCCA) is the most common type of CCA in IBD patients (RR = 2.61; 95% CI, 1.72–3.95) [49]. The major risk factor for CCA is the development of primary sclerosing cholangitis (PSC) (HR 28.46) [50]. Other risk factors are older age, male sex, duration of IBD, history of colorectal cancer (CRC), or colonic dysplasia [10]. CCA results in a poor prognosis and the surveillance strategy for patients with IBD and PSC should include magnetic resonance cholangiopancreatography (MRCP) annually, serum CA 19-9 testing periodically, and annual colonoscopy as they also have a higher risk of CRC [14].

Four population-based studies demonstrated a higher risk of liver cancer (SIR 15.3; 95% CI, 5.6–33.2), which seems to be particularly higher in patients with a first hospitalization for IBD at a very young age [20,23,24,40].

Three studies found an association between IBD and pancreatic cancers (HR 1.43; 95% CI, 1.30–1.58), especially in IBD patients with PSC (HR 7.55; 95% CI, 4.94–11.5) [45]. Patients with IBD did not differ in cancer stage or pancreatic cancer mortality when compared to healthy controls [40,41,45]. Yuan et al. performed a population-based, nested case-control study to examine the associations between autoimmune conditions and pancreatic cancer risk in patients aged ≥66 years within the Surveillance, Epidemiology, and End Results Program (SEER)-Medicare population and found an association for both PSC with pancreatic ductal adenocarcinoma (PDAC) (OR = 1.37; 95% CI, 1.18–1.59) but also UC (OR = 1.18; 95% CI, 1.07–1.31). UC was also associated with an increased risk of pancreatic neuroendocrine tumor (OR = 1.78; 95% CI, 1.17–2.70) [51]. However, further studies are needed to confirm this finding.

As to the risk of developing skin cancers, nine population-based studies found an association between IBD and NMSC, with IRR = 1.46 (95% CI, 1.40–1.53) [17,19,28,29,31,39,41,42,44]. One population-based study, one retrospective study, and one case-control study reported an increased risk of melanoma in patients with IBD than in healthy controls (IRR, 1.29; 95% CI, 1.09–1.53) [28,31,41,46]. Finally, some studies have pointed to the role of immunomodulating therapies—especially thiopurines in developing EICs [28,44,48,52].

Regarding female reproductive cancers, three population-based studies reported an increased risk of breast cancer in patients with IBD (UC: SIR = 2; 95% CI, 1.12–3.34; and CD: SIR = 2.42; 95% CI, 0.98–5.03), especially 8–10 years after IBD diagnosis [23,24,38,42]. Conversely, one study found a decreased risk of breast cancer in IBD (SIR 0.11; 95% CI, 0.00–0.64) [34]. Taborelli et al. found that patients with UC had an increased risk of corpus uteri cancer (SIR = 2.67, 95% CI: 1.07–5.50) [44]. Some studies demonstrated that patients with IBD were at increased risk of cancer of the cervix uteri (UC: SIR = 5.7; 95% CI, 2.4–11; and CD: IRR = 1.53; 95% CI, 1.04–2.27) and cervical dysplasia (SIR = 1.65; 95% CI, 1.10–2.37) [22,30,32,40,47]. In contrast, three studies did not find any association between IBD and cervical abnormalities (OR = 1.03; 95% CI, 0.77–1.38) [27,33]. There are more abnormal cervical smears in IBD patients with a concomitant smoking habit [27]. Finally, Singh et al. described an association between CD and cervical abnormalities only in patients exposed to more than 10 prescriptions of oral contraceptives (OR, 1.66; 95% CI, 1.08–2.54) [25].

Five studies reported an increased risk of prostate cancer, especially in UC (SIR = 2.47; 95% CI, 1.24–4.95) [23,30,39,40,41,43]. This risk does not appear to be dependent on older age or prostate-specific antigen (PSA) values [43]. In contrast, Jussila et al. found a lower risk of prostate cancer in UC [29]. In a recent metanalysis that included 18 cohort studies with 592,853 participants, the authors showed an elevated risk of incident prostatic cancer in UC patients (HR = 1.20; 95% CI, 1.06–1.38) but not Crohn’s patients (HR = 1.03; 95% CI, 0.91–1.17) [53]. Other urological malignancies, including kidney cancer, bladder cancer, and cancers of the urinary tract, were also found to be more frequent in IBD than in healthy controls [18,24,39,41,44]. Only one study found a higher risk of testis cancer in patients with CD than in the general population (SIR = 2.28; 95% CI, 1.39–3.53) [24].

Five studies demonstrated a higher risk of respiratory malignancies (OR = 1.19; 95% CI, 1.07–1.32), such as cancers of the lungs, bronchi, or trachea [18,30,38,42,48]. In contrast, two studies did not find an association between IBD and lung cancer [38,39]. 

Thyroid cancer was also more frequent in IBD patients (SIR = 1.93; 95% CI, 1.28–2.79) [29,37,40,44]. Older age was shown to be a protective factor in the development of thyroid cancer [37].

An increased risk of central nervous system (CNS) and brain tumors was also demonstrated, especially for CD (SIR = 5.08; 95% CI, 1.64–15.76) [17,18,39,40]. In one study, this risk was associated with extensive ulcerative colitis at diagnosis [17].

In conclusion, in a meta-analysis by Pedersen et al. evaluating the risk of EICs in IBD patients and including eight population-based cohort studies for a total of 17,052 IBD patients, the authors did not find a higher risk of overall EICs (SIR = 1.10; 95% CI, 0.96–1.27); however, CD patients had an increased risk of lung cancer (SIR = 1.82; 95% CI, 1.18–2.81), urinary bladder cancer (SIR = 2.03; 95% CI, 1.14–3.63), and skin cancer (SIR = 2.35, 95% CI, 1.43–3.86), while UC patients had an increased risk of hepatobiliary cancer (SIR = 2.58, 95% CI, 1.58–4.22) and leukemia (SIR = 2.00, 95% CI, 1.31–3.06) but a decreased risk of pulmonary cancer (SIR = 0.39, 95%CI, 0.20–0.74) [15]. Conversely, another meta-analysis of Lo et al., including 15 studies, showed that the overall risk of EICs was increased in both CD (IRR = 1.43; 95% CI, 1.26–1.63) and UC (IRR = 1.15; 95% CI, 1.02–1.31) compared to the healthy controls. Particularly, in patients with IBD, the risk was higher for skin cancer (IRR = 2.22; 95% CI, 1.41–3.48 and IRR = 1.38; 95% CI, 1.12–1.71, respectively) and hepatobiliary (IRR = 2.31; 95% CI, 1.25–4.28 and IRR = 2.05; 95% CI, 1.52–2.76, respectively) malignancies. Moreover, patients with CD had also a higher risk of lung cancer (IRR = 1.53; 95% CI, 1.23–1.91) [16].

Except for CCA, there is no evidence for prevention and early diagnoses of EICs in patients with IBD, so clinicians caring for IBD patients should encourage them to follow the same surveillance programs as the healthy population, based on individual risks and clinical judgment [10].

**Figure 1:**
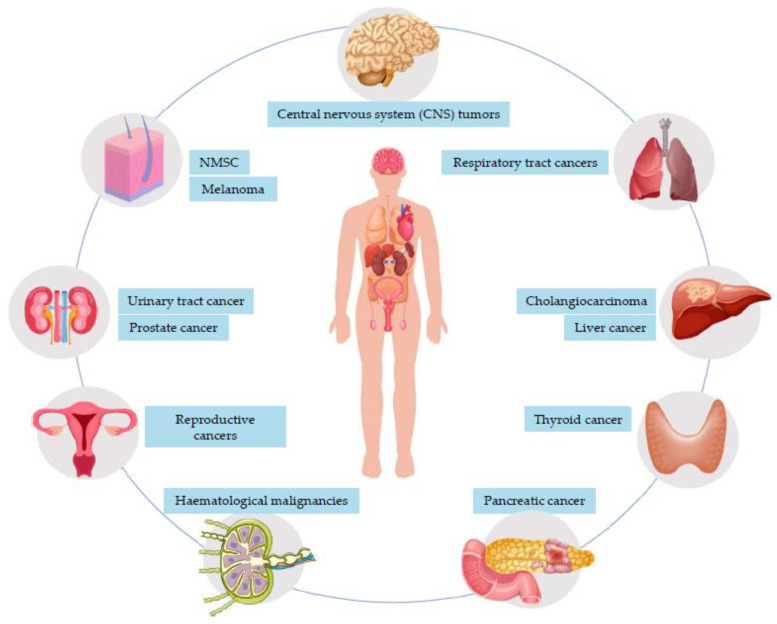
Summary of IBD and risk of malignancies.

Figure 1 summarizes the association between malignancies and IBD.

#### 3.1.2. Hematological Malignancies

Several population-based studies have suggested an increased risk of hematological malignancies in patients with IBD compared with the general population [19,21,29,30,31,34,35,39,40,54,55,56,57,58,59,60,61]. On the other hand, other studies, such as the one reported by Lewis et al., did not support this association (RR = 1.20; 95% CI, 0.67–2.06) [62].

Lymphoma, specifically non-Hodgkin lymphoma (NHL), appears to be the most observed hematological malignancy in IBD patients, especially in male CD patients. However, in some population cohorts, the incidence of lymphoma is also elevated in UC patients [29,56,63]. Lo et al., in their meta-analysis published in 2021 including 15 population-based studies, found a significantly increased risk of hematologic malignancies (IRR = 2.40; 95% CI, 1.81–3.18), especially lymphomas (IRR = 1.86; 95% CI, 1.04–3.32), among patients with CD. The relative risk reported in this study was smaller compared to that reported in previous investigations [16]. A recent meta-analysis that included 36 studies, with a total of 617,386 patients, found that reported incidence rates for lymphoma in IBD ranged from 0.0/100,000 person-years (py) (95% CI, 0.0–3.7/100,000) to 89/100,000 py (95% CI, 36–160/100,000) [64]. When the incidence rates were calculated based on the disease type, incidence rates of lymphoma in CD ranged from 0.0/100,000 py (95% CI, 0.0– 3.7/100,000) to 91/100,000 py (95% CI, 18–164/100,000), while for UC it ranged from 0.0/100,000 py (95% CI, 0.0–3.7/100,000) to 95/100,000 py (95% CI, 0–226/100,000) [64]. Both these metanalyses included data from patients with a history of previous or concomitant therapy with immunomodulators and biological drugs.

The estimated relative risk may differ between studies, but it is worth pointing out that the absolute risk of developing hematological malignancy in IBD patients remains relatively low and the overall incidence is still considered rare [64]. While the role of medical therapy, including thiopurines and Tumor Necrosis Factor (TNF)-α Inhibitors (TNFi), as a risk factor for hematological malignancy is supported by recent data, it remains unclear whether the disease itself poses a risk of developing these tumors. Indeed, determining whether the increased rate of hematological malignancies is related to the severity and duration of IBD, the concomitant administration of immunomodulators and biologics, and the interplay between these factors, represents a challenge [20,57,65].

A recent study conducted in Sweden and Denmark by Olén et al. investigated a population cohort consisting of 164,716 patients diagnosed with IBD between 1969 and 2019, who were matched with 1,639,027 individuals from the general population [60]. The findings revealed that both CD patients (hazard ratio, HR = 1.32; 95% CI, 1.16–1.50) and UC patients (HR = 1.09; 95% CI, 1.00–1.20) had a higher likelihood of being diagnosed with lymphoma [60]. However, the increase in lymphoma cases over 10 years was minimal, even among CD patients (0.08%; 95% CI, 0.02–0.13) [60]. Childhood-onset and colonic location of the disease in patients with CD, and extensive colitis and/or concomitant PSC in patients with UC, were associated with a higher risk of developing lymphoma [60]. Moreover, in their analysis, the authors also examined the HRs of lymphoma during the period from 2003 to 2019, finding an increased risk in the past two decades. These findings suggest that the elevated utilization of immunomodulators and biologics in CD might account for this disparity [60].

A population-based cohort study from the Eneida registry (nationwide study on genetic and environmental determinants of inflammatory bowel disease) was conducted involving 21,740 patients diagnosed with IBD and identifying 52 cases of lymphoma (2.39 lymphoma cases per 1000 IBD patients). The majority of these cases were observed in patients with UC [66].

A wide histological variety of lymphomas has been described in patients with IBD, mainly EBV-associated lymphomas, including some rare but aggressive lymphomas such as hepatosplenic T-cell lymphomas or plasmablastic lymphoma [67,68]. In the recent population-based cohort study conducted in Sweden and Denmark, the authors identified increased HRs for aggressive B-cell non-Hodgkin lymphoma (NHL) in both CD and UC patients, including a particularly pronounced risk of T-cell NHL in CD patients (HR = 2.91; 95% CI 1.87–4.53), although they did not consider EBV serology [60].

The limited available data seem to show that the prognosis of lymphoma in patients with IBD seems to be the same as for de novo presentation [63,68,69]. A French cohort study including 52 lymphoma patients with IBD recently reported that progression-free survival (PFS) at 3 years in patients with IBD and lymphoma is comparable to that of patients with de novo lymphoma, as reported by the literature (PFS at 3 years was 85% in the overall population with no difference between exposed or unexposed patients to thiopurines and/or TNFi) [68].

The association between thiopurines and other types of cancer is still controversial [30,34,36,54,65,70,71,72,73,74,75,76,77,78,79,80].

Primary Intestinal Lymphoma (PIL) is a rare form of NHL that primarily affects the gastrointestinal tract, specifically the small intestine [81]. While PIL can occur in individuals without any underlying conditions, there is a notable association between PIL and IBD, particularly CD [81,82,83,84]. Four case series described this association [81,82,83,84]. Phillips et al. reported on fifteen IBD patients with PIL treated in eight different centers [81]. In this case series, PIL mainly occurred in middle-aged men (80%), with an average age of 47.8 years at diagnosis [81]. Notably, two-thirds of patients had a history of thiopurines or TNFi use, and two-thirds developed PIL in the luminal site affected by IBD [81]. In the CESAME (*Cancers et sur-risque associé aux maladies inflammatoires intestinaliss en France*) population, the incidence of PIL was 0.12/1000 patient-years, with a corresponding SIR of 17.51 (95% CI, 6.43–38.11) [82]. The individuals who were exposed to thiopurines faced the greatest risk; it is believed that the Epstein-Barr virus (EBV) is often involved in these cases [67,82].

There have been suggestions of a slightly higher occurrence of leukemia in patients with IBD [15,85]. However, when examining large population-based studies, the findings have been inconsistent [57,86,87,88]. Particularly, some studies have reported a small but significant increase in leukemia risk [87,88], while others have found no significant association [20,23,58]. These discrepancies could be attributed to variations in the duration of follow-up periods and the average age of the analyzed group.

A large Swedish population-based study of 47,679 IBD patients found that patients with UC had an increased risk of acute myeloid leukemia (AML) (SIR = 2.53; 95% CI, 1.2–4.8), but the authors did not specifically evaluate the role of thiopurines [86]. Conversely, a nationwide population study in Taiwan, consisting of 3348 IBD patients without a history of cancer, found a notably elevated relative risk of leukemia but with a wide confidence interval (SIR = 19.23; 95% CI, 6.2–44.9) [57].

Based on the available evidence, there is a low probability of IBD contributing to an elevated risk of chronic lymphocytic leukemia [60]. A population-based control case–control study using data from the U.S. Surveillance Epidemiology and End Results (SEER)-Medicare database was designed to determine the risk of myeloid malignancies in patients over 67 years of age with a history of autoimmune diseases [89]. The risk was increased in patients with UC (OR = 1.72; 95% CI, 1.28–2.31) but not in those with CD [89].

There appears to be a moderately increased risk of developing myeloma in individuals with IBD compared to the general population, especially in UC patients [90]. In fact, a recent U.S. cross-sectional cohort analysis included 1750 patients with UC and showed increased overall odds of having Multiple Myeloma (OR = 1.26; 95% CI, 1.04–1.58) [90].

Whether the prognosis of hematological malignancies, particularly lymphoma, is different between the IBD population and the general population is unknown [10]. 

### 3.2. Safety of Immunomodulators and Biologic Agents

Currently, there are various drugs available for treating IBD, including immunomodulators (such as thiopurines and methotrexate (MTX)), biologic agents (such as TNFi, anti-integrin, and anti-IL12/23), as well as more recent options including Janus-kinase inhibitors and SP1 modulators [91]. The effectiveness of these treatments has been demonstrated to varying extents in randomized clinical trials, and they are recommended in clinical guidelines [92,93]. Determining the cancer risk related to IBD therapy poses challenges due to the difficulty of distinguishing the risk of the therapy itself from that of the underlying disease and other potential influencing factors [34]. Additionally, it is complicated to estimate the association with a specific medication because patients are exposed to different drugs throughout their disease course [16]. Despite these complexities, there is evidence suggesting that certain treatments for IBD may increase the risk of developing specific cancer types [78,94]. Moreover, it is important to note that newer drugs have not had sufficient opportunity to gather substantial real-world data and, unfortunately, trials do not have adequate numbers of patient-years to conclude a clear association between the risk of malignancy and the use of these drugs [95]. In this case, we may consider the existing evidence from other inflammatory conditions with a larger pool of patient experience to make informed decisions about potential risks [96]. However, it is important to bear in mind that this approach assumes comparable levels of cancer risk and immunosuppressive effects between inflammatory-mediated disorders, and it may not directly apply to dosing regimens specific to IBD [10,91,92,93,97,98].

Currently, I-CARE (Inflammatory Bowel Disease Cluster for Assessment of Risk and Epidemiology) is an ongoing investigator-initiated observational European prospective cohort study that aims to provide valuable insights into the long-term benefits and risks associated with biological therapies in patients with IBD [99]. 

**Figure 2:**
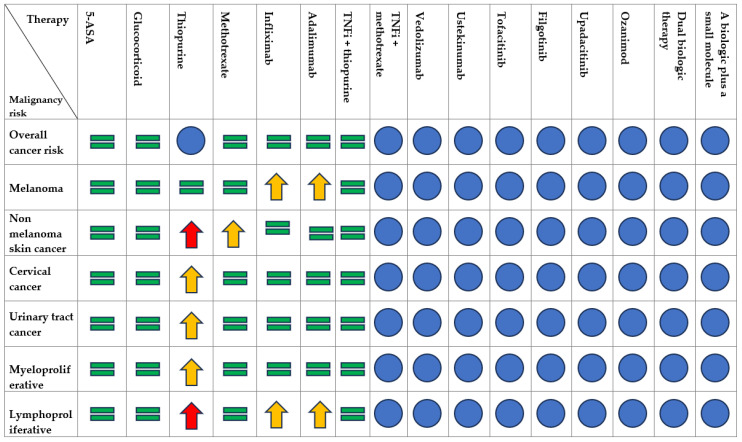
Summary of IBD therapies and risk of malignancies according to current evidence. In red, strong evidence of risk of malignancy. In yellow, weak or moderate evidence of increased risk. In green, no evidence of increased risk. In blue, insufficient evidence. Abbreviations: 5-ASA, 5-aminosalicylate; TNFi, TNFα-inhibitors.

Figure 2 summarizes the risk of developing neoplasms associated with immunomodulators or biological therapy.

#### 3.2.1. Thiopurines

Thiopurines and their potential association with cancer have been investigated in several studies [70,72,79,94,100,101,102,103,104,105,106,107,108,109]. While the results have been somewhat conflicting, the overall consensus suggests a moderately increased risk of certain types of cancers, particularly lymphoproliferative disorders [70,100], such as EBV-related lymphomas and hepatosplenic T-cell lymphoma, and NMSC [108]. The risk appears to be dose-dependent, with higher cumulative dosages and a longer duration of thiopurines use being associated with elevated cancer risk [101]. 

In fact, thiopurines have been linked to EBV-related and non-EBV-related lymphomas, but the association with the former is more significant [60,63]. In a recent retrospective cohort study originating from the population-based IBD South-Limburg (IBDSL) cohort, the incidence of lymphoma in IBD patients treated with thiopurines was 1.04 per 1000 py [103]. The risk seems reversible after drug withdrawal, as described by the CESAME (*Cancers et sur-risque associé aux maladies inflammatoires intestinaliss en France*) group in 2009 [70] and as highlighted by a meta-analysis of 18 studies published in 2014, in which the overall SIR for lymphoma was 4.49 (95% CI, 2.81–7.17) and reverted to baseline after discontinuation (SIR: 1.42; 95% CI, 0.86–2.34) [110]. A recently published meta-analysis of high-quality observational studies confirmed that IBD patients exposed to thiopurines monotherapy are at increased risk of lymphoma when compared with IBD patients unexposed to TNFi or thiopurines (IRR = 2.23; 95% CI, 1.79–2.79) [111].

Therapy with thiopurines in patients seropositive with EBV has been associated with Epstein-Barr-virus (EBV)-associated B cell lymphoma, which is unpreventable during use, strongly increases with age, and is doubled in men, but which is reversible on drug withdrawal [34,70,100,101,110,112,113,114].

In EBV-seronegative patients undergoing therapy with thiopurines, the risk of a primary infection with EBV has been associated with early post-mononucleosis lymphomas [70], mainly occurring in young male patients, and hemophagocytic syndrome [115,116,117,118]. The latest risk may also be present in primary CMV infection.

Secondary hemophagocytic lymphohistiocytosis (HLH) is a rare and life-threatening syndrome known for excessive inflammatory reaction and tissue destruction [119]. HLH can be triggered by a viral infection, especially CMV and EBV, or malignancy [116]. A recent population study included data from hospitalized patients in a nationwide inpatient sample and confirmed a higher incidence of HLH in IBD patients [119]. The recent systematic reviews by Li et al. and Brambilla et al. on HLH in IBD patients confirmed that the majority of cases are seen in people receiving therapy including thiopurines [116,118]. A large study performed with 1483 patients over 17 years old highlighted that the risk of a primary infection with EBV and the associated complications in patients undergoing therapy with thiopurines, such as hemophagocytic syndrome and/or lymphoma, are present also in older patients [120].

The recent ECCO guidelines on malignancy recommended caution when considering thiopurines in young male patients [<35 years] who are EBV naïve [10,78].

In a recent large U.S. population study cohort, including 56,314 IBD patients from the Veteran Affairs dataset, current thiopurines use was associated with an increased risk of acute myeloid leukemia/myelodysplastic syndrome (AML/MDS), which reverted to normal after the discontinuation of the drug [101], theoretically explained by the proliferation of blood cells with defective DNA-mismatch repair that escape the cytotoxic effect of drugs [121].

IBD by itself is suspected to promote skin cancers and, therefore, sun protection and skin surveillance are recommended from diagnosis in all patients [10]. As previously mentioned, patients on thiopurines treatment have an increased risk of NMSC, but not melanoma [28,106,107,108,109]. In fact, in a recent retrospective study based on the University of Manitoba IBD Epidemiology Database, which included 11,228 IBD cases and 104,725 matched controls, therapy with thiopurines was found to increase the risk of basal cell carcinoma (BCC) (HR = 1.52; 95% CI, 1.10–2.10) and cutaneous squamous cell carcinoma (cSCC) (HR = 7.81; 95% CI, 4.56–11.05) but not melanoma (HR = 1.51; 95% CI, 0.50–4–54) [122].

A meta-analysis published in 2018 and including 13 studies for a total number of 149,198 patients suggested that patients with IBD treated with thiopurines had a significantly increased risk of developing NMSC (RR = 1.88; 95% CI, 1.48–2.38] [109]. The risk of NMSC seems to increase depending on the total cumulative thiopurines dose [105,108] and decrease [106], or return to baseline [105,123], after discontinuing therapy, although additional data are needed to support this trend. However, the risk of NMSC attributable to thiopurines is difficult to interpret since studies are heterogeneous and many factors involved in the risk of skin cancer are not considered (sun exposure and skin phenotype, among others).

There is conflicting data on the risk of cervical neoplasia in women with IBD, particularly those undergoing thiopurines [28,47,70,124,125,126]. In a recent meta-analysis of five unselected population-based studies including 74,310 patients with IBD and 2,029,087 reference patients, the authors did not show a statistically significant increased risk for cervical cancer in IBD patients under therapy with thiopurines (HR = 0.96; 95% CI, 0.60–1.50) [127]. However, a meta-analysis published in 2015 that took into account five retrospective cohort studies and three case controls studies of patients with IBD on any immunosuppression, including steroids, thiopurines, or biologic therapy, with cervical high-grade dysplasia/cancer, revealed an increased risk of cervical high-grade dysplasia (HGD) or cancer in patients with IBD on any immunosuppressive therapy and compared with population controls without IBD (OR: 1.34; 95% CI: 1.23–1.46] [125].

Large studies taking into account different potential confounding factors such as smoking, Human Papilloma Virus (HPV) status, HPV genotype, and the variability of outcomes, such as cervical dysplasia, cervical carcinoma, or cervical abnormalities, are lacking [128]. In this unclear scenario, the recent ECCO guidelines on malignancy suggested that women on thiopurines therapy participate in screening programs available for the general population [10].

Concerning urinary-tract neoplasms, there is some evidence regarding the association with thiopurines use in IBD patients, although the risk is almost completely restricted to older males, particularly smokers [77,94,129]. This trend was not confirmed in two retrospective cohort studies [73,130]. 

In the CESAME cohort (*Cancers et sur-risque associé aux maladies inflammatoires intestinaliss en France*), active but not previous thiopurines therapy increased the risk of urinary-tract neoplasms compared with no treatment (HR = 2.82; 95% CI, 1.04–7.68; *p* = 0.04) [94]. Smoking status was not recorded in this study, but increasing age was also a significant risk factor (HR = 13.26; 95% CI, 3.52–50.03) [94]. However, in a recently published Dutch real-world study including 1016 IBD patients with 9860 py of follow-up undergoing thiopurines monotherapy, the incidence rate of urinary neoplasms under treatment was lower than in the CESAME cohort (0.21/1000 py) [103]. 

#### 3.2.2. Methotrexate

Although evidence is scarce and derived from case-control and cohort studies involving a small number of patients, there is a potentially higher risk of NMSC in patients receiving MTX treatment [28,131,132,133]. However, a substantial cohort study utilizing data from the Spanish ENEIDA registry (a nationwide study investigating genetic and environmental factors of inflammatory bowel disease) with a median follow-up of 98 months, did not find an elevated risk of extracolonic cancer in individuals with IBD undergoing MTX monotherapy [76].

The utilization of MTX has raised concerns about its potential to heighten the likelihood of developing skin cancer [123]. In a significant nested case-control study involving over 800 patients exclusively treated with MTX, it was discovered that MTX exposure was linked to a higher risk of NMSC (OR = 8.55; 95% CI: 2.55–31.8). The study identified five cases of NMSC among patients exposed to MTX for less than one year, but this correlation was not observed in patients exposed for a duration of thirteen months or longer [123].

In a recent Danish case-control study, the use of MTX for any disease was found to be associated with an increased risk of BCC, cSCC, and melanoma among the IBD population. However, it should be noted that data from other diseases cannot be directly applied to the IBD population. The study reported adjusted odds ratios (oRs) of 1.29 (95% CI, 1.20–1.38), 1.61 (95% CI, 1.37–1.89), and 1.35 (95% CI, 1.13–1.61) for BCC, cSCC, and melanoma, respectively. Furthermore, the risk of BCC and cSCC increased with higher cumulative doses of MTX [134]. It is important to consider additional factors such as skin type and skin protection in the development of NMSC, although these factors were not included in the analysis [134].

#### 3.2.3. TNFα Inhibitors

Numerous studies have examined the potential cancer risks linked to TNFi, considering their extensive usage in treating IBD [79,104,135,136,137]. The available evidence indicates that the overall risk of malignancies in IBD patients treated with TNFi drugs is not increased and comes from both clinical trials and real-world data.

Multiple meta-analyses reported no increased overall risk of cancer in IBD patients with TNFi [63,74,138,139,140]. However, due to the frequent simultaneous use of thiopurines and challenges in accounting for confounding factors such as disease severity or patient characteristics, some uncertainties persist. Particularly, the risks associated with specific types of cancer, mainly lymphoma and melanoma, raise some unresolved questions [79,104,111].

Regarding overall cancer risk in IBD patients, a systematic review and meta-analysis published in 2016 analyzed as a secondary outcome the malignancy risk associated with biological therapies (adalimumab (ADA), certolizumab, golimumab, infliximab (IFX), natalizumab, or vedolizumab (VDZ)) in IBD and included data from 23 randomized placebo-controlled studies (9455 patients) reporting at least a single incident cancer. They showed no association between biologics and cancer risk in patients with IBD (OR = 0.90; 95% CI, 0.54–1.50); however, data were scarce, and the periods of exposure and follow-up were too short to permit valid conclusions. In fact, the follow-up periods were up to 24 months, a period considered insufficient to evaluate the incidence of new malignancies [141]. 

In 2021, Muller et al. published a systematic review assessing the risk of cancer in adult IBD patients exposed to TNFi in observational cohort studies [136]. Out of the 20 studies included, 14 studies focused on patients with IBD who received TNFi monotherapy without being exposed to thiopurines [73,75,100,142,143,144,145,146,147,148,149,150]. In this subgroup of patients, a total of 189 cases of malignancy were observed out of 52,198 patients exposed to TNFi, resulting in an overall occurrence rate of 0.4% [136]. It is important to note that these studies had varying follow-up periods, ranging from 7 to 80 months, and some studies did not provide this information [136]. Among the eleven studies analyzed, ten of them did not discover a noteworthy correlation between the usage of TNFi and the risk of cancer [75,79,100,123,139,144,148,150,151,152,153]. Nevertheless, a study indicated a notably increased likelihood of lymphoma in patients who underwent TNFi treatment when compared to those who did not receive the medication (adjusted HR= 2.41; 95% CI, 1.60–3.64; *p* < 0.001) [100].

In addition, some evidence did not demonstrate an increased cancer risk associated with TNFi in elderly IBD patients [138,140,151]. The study conducted by Cottone et al. examined the potentially harmful effects of biologics in elderly patients with IBD through a case-control analysis [151]. This study compared the incidence of malignancy in a group of patients with IBD over 65 years old treated with TNFi (*n* = 95), a group of patients with IBD over 65 years old not receiving TNFi (*n* = 190), and a group of patients 65 years old or younger treated with TNFi (*n* = 190) [151]. There were two cases (3%), four cases (2%), and no cases of cancers, respectively, in the groups: elderly cohorts had increased cancer incidence, but no impact of biological therapy on cancer development was observed in these cohorts [151].

Two meta-analyses also showed that the overall cancer risks in IBD patients older than 60 years of age were not increased by exposure to TNFi [138,140]. The systematic review and meta-analysis by Piovani et al. included nine studies reporting at least one case of malignancy and showed that the overall cancer risk in IBD patients older than 60 years of age was not increased using TNFi (OR = 0.90; 95% CI, 0.64–1.26). Nevertheless, even though the majority of the included studies had a follow-up duration exceeding 3 years, they were unable to completely rule out the possibility of a slight rise in risk due to the relatively brief duration of exposure in some cases [140].

Concerning hematological malignancies, in some observational studies and meta-analyses, TNFi has been associated with an increased risk of lymphoma [80,100,111,113], while others did not confirm these findings [63,154,155]. 

A meta-analysis by Chupin et al. of observational studies aiming to assess the comparative risk of lymphoma with TNFi and/or thiopurines in IBD found thirty-three lymphomas diagnosed in 80,987 py in patients exposed to TNFi monotherapy, yielding an incidence rate of 0.41 per 1000 py (95% CI, 0.29–0.57). Comparison of the risk of lymphoma in patients exposed to TNFi monotherapy and patients unexposed to TNFi or thiopurines was based on four studies and the pooled IRR of lymphoma was 1.52 (95% CI, 1.06–2.19; *p* = 0.023).

A French nationwide cohort study by Lemaitre et al. including 189,289 patients from the French National Health Insurance claim database known as SNIIRAM (*Système National d’Information Interrégimes de l’Assurance Maladie*) followed up for a median of 6.7 years recently suggested that the risk of lymphoma was increased two-fold in patients exposed to TNFi alone and six-fold in those exposed to combination therapy with thiopurines [100]. Particularly, they reported 32 cases of lymphoma in 30,294 patients exposed to TNFi monotherapy (incident rate, IR = 0.41; 95% CI, 0.27–0.55) [100]. In a multivariable Cox model, compared with unexposed patients, the risk of lymphoma was higher among those exposed to TNFi monotherapy (HR = 2.41; 95% CI, 1.60–3.64; *p* < 0.001) [100].

On the contrary, other studies have presented reassuring safety findings. For instance, an examination of the Quebec Claims database, which involved 19,582 patients, did not discover any heightened risk of lymphoma among patients exposed to TNFi [123].

The PYRAMID registry was specifically established to explore the risk of lymphoma in patients with CD who were exposed to ADA. The registry included a total of 5025 patients, with a follow-up period spanning 16,689 patient-years. The results demonstrated a lower lymphoma rate [10 cases] compared to the estimated rate in the general population [156]. The observed lymphoma rate (0.060 E/100 py) was lower than the estimated background rate (0.084 E/100 py), and the upper limit of the one-sided 95% confidence interval for the observed rate (0.102 E/100 py) was lower than twice the estimated rate (0.168 E/100 py) [156].

Similarly, the ENCORE registry, which involved 1541 patients with CD treated with IFX, found no association between IFX and lymphoproliferative disorders or malignancies [152]. The prospective, observational ENCORE registry, spanning five years, revealed no excess risk of lymphoma among patients diagnosed with IBD who were solely treated with TNFi [152].

Finally, the REFURBISH study examined reports submitted to the Food and Drug Administration’s adverse event reporting system as well as Medline search. The study indicated that there was no increased risk of T-cell non-Hodgkin lymphoma associated with TNFi monotherapy compared to combination treatment or thiopurines monotherapy [135].

Limited data suggest that patients with IBD who develop lymphoma while receiving TNFi have a similar oncological prognosis as those with lymphoma and IBD, but no history of TNFi exposure [136]. To investigate this further, a multicenter retrospective cohort analysis was conducted in seven French tertiary centers from 1999 to 2019, involving fifty-two patients, thirteen of whom had been exposed to TNFi [68]. The findings indicated that the prognosis for lymphomas occurring in IBD is generally favorable and comparable to the expected outcome, regardless of exposure to biologics and/or immunomodulators [68].

According to a retrospective cohort study published in 2021, there does not appear to be an increased risk of acute myeloid leukemia and myelodysplastic syndrome associated with TNFi in patients with IBD from the Veteran Affairs data set (HR = 0.86; 95% CI, 0.34–2.19, *p* = 0.7462) [101].

The role of TNFi in the risk of cSCC, BCC, and melanoma in patients with IBD has been examined [157]. Studies on this subject matter often face the limits imposed by inadequate statistical power and confounding factors, such as prior or concomitant exposure to thiopurines, age, sex, UVR exposure, exposure to PUVA therapy, and skin phototype [157].

In 2012, a study analyzing a health insurance claims database was conducted, involving 108,579 individuals with IBD who were each matched to four controls without IBD [28]. The findings of this study suggested that the use of biologics, specifically TNFi and natalizumab, was linked to a notable increase in the risk of melanoma among the IBD population (OR = 1.88; 95% CI, 1.08–3.29). Notably, the increased risk was primarily observed in individuals with Crohn’s disease (OR = 1.94; 95% CI, 1.03–3.68), whereas no significant association was found in those with UC (OR = 1.73; 95% CI, 0.53–5.63) [28].

However, this finding has not been replicated in other studies [63,139,157]. A systematic review and meta-analysis published in 2020, which included 7901 patients with IBD exposed to TNFi and 135,370 biologicIe patients, did not reveal a statistically significant association between treatment with TNFi and melanoma in patients with IBD (RR = 1.20; 95% CI, 0.60–2.40) [139,157,158].

Several studies reported no association between NMSC and TNFi use in IBD [28,79,136,159]. A recent systematic review that included 28 studies and 298,717 IBD patients reported 692 (1%) malignancies in patients with IBD exposed to TNFi [136]. NMSCs were the most frequently observed malignancies (123/692; 17.8%) and were reported at the same rates as expected in the general non-IBD population [77,136].

#### 3.2.4. Combination Therapy of TNFi with Immunomodulators

Combination therapy is considered effective and is suggested for moderate-to-severe CD in recent guidelines [93,160,161]. There is also evidence for its use in moderate-to-severe UC patients [92,162]. In clinical practice, it is crucial to strike a balance between the potential advantages of enhanced effectiveness and reduced immunogenicity associated with combination therapy, and the possibility of heightened long-term adverse events [92,93]. It is worth noting that, thus far, there have been no safety concerns observed in the short term [100,163]. It is crucial to personalize the evaluation of risks, considering that certain patient populations, such as the elderly, might face an increased susceptibility to infections or lymphoma, while young males could be at a higher risk for specific complications, such as hepatosplenic T-cell lymphoma [78]. There is a lack of adequate data regarding the risk of lymphoma in individuals with IBD who are exposed to a combination of TNFi and MTX [10].

According to the available evidence, there is no added risk of developing solid-organ or skin cancers (such as melanoma and NMSC) among patients with IBD who undergo combination therapy (using TNFi and thiopurines or MTX) compared to those who receive either TNFi or thiopurines in monotherapy [74,139,164]. Nevertheless, the likelihood of developing lymphoma due to the combined use of TNFi and thiopurines treatment is considerably greater compared to using either thiopurines or TNFi alone [100,135,154]. Specifically, the risk of hepatosplenic T cell lymphoma significantly rises with the administration of both TNFi and thiopurines [165]. Non-EBV-related hepatosplenic T-cell lymphomas [78,110] are usually fatal but are extremely rare and occur in male patients younger than 30 years with CD who have been receiving combination therapy with thiopurines and TNFi for longer than 2 years [165].

A meta-analysis of four observational studies by Chupin et al., including 261,689 patients with IBD, assessed the risk of lymphoma in four comparator groups (combination therapy with TNFi plus thiopurines, TNFi monotherapy, thiopurines monotherapy, and unexposed to TNFi or thiopurines) [66]. They found a higher pooled incidence rate ratio (IRR) per 1000 py for patients in combination therapy as compared with patients unexposed to both drugs (IRR = 3.71; 95% CI, 2.30–6.00; *p* ≤ 0.01) than those exposed to TNFi monotherapy or thiopurines monotherapy alone [66]. Eighteen lymphomas were diagnosed in 17,620 py in patients exposed to combination therapy, yielding an incidence rate of 1.02 per 1000 patient-years (95% CI, 0.64–1.62) [66].

An analysis of the Quebec Claims database already previously mentioned, including 19,582 patients, only showed an increased risk for lymphoma in patients with combination treatment (OR = 8.64; 95% CI, 1.33–56.06), although exposure to TNFi in this cohort was very low [123].

In their recently published study, Olén and colleagues investigated a population cohort consisting of 164,716 patients diagnosed with IBD between 1969 and 2019 matched to 1,639,027 individuals from the general population, finding that IBD patients had a higher likelihood of being diagnosed with lymphoma and that the highest HRs were observed in patients exposed to combination therapy for both CD (HR = 2.58; 95% CI, 1.48–4.48) and UC (HR = 3.41; 95% CI, 2.05–5.69) [60].

In a French, nationwide, insurance database study published in 2017, the risk of lymphoma was higher among patients exposed to thiopurines monotherapy (HR = 2.60; 95% CI, 1.96–3.44), TNFi monotherapy (HR = 2.41; 95% CI, 1.60–3.64), or combination therapy (HR = 6.11; 95% CI, 3.46–10.8) [100].

Although initial data analysis from clinical trials of ADA in CD indicated that patients who received combination therapy with thiopurines or MTX had a higher risk of both NMSC and all other cancers compared to patients treated with ADA alone [166], this observation was not supported by subsequent post-marketing registries, PYRAMID for ADA and TREAT (Crohn’s Therapy, Resource, Evaluation, and Assessment Tool) for IFX [156,164]. In these registries, the rates of malignancies, excluding lymphoma, did not show statistically significant differences between CD patients receiving TNFi with or without concurrent thiopurines therapy at the beginning of treatment [162].

#### 3.2.5. Anti-Integrins

Despite the limited availability of long-term follow-up and real-world evidence, comprehensive safety analyses of clinical trials, open-label extension studies, observational studies, and meta-analyses have not identified a significant rise in the risk of solid-organ or hematologic malignancies in VDZ-treated IBD patients [167,168,169,170,171,172].

In the GEMINI long-term safety study, there was no apparent correlation found between the occurrence of malignancies and factors such as age, sex, type of malignancy, or duration of VDZ exposure. Basal cell carcinoma was the most frequently observed malignant neoplasm, with an incidence of less than 1% in both UC and CD. The rate of malignancies at all sites was 9.8/1000 per year in UC and 8.3 in CD, indicating that the overall occurrence of malignancies does not appear to be affected by VDZ [168].

The Vedolizumab Global Safety Database has reported post-marketing surveillance data, which includes over 4 years of follow-up for patients with CD or UC undergoing VDZ treatment [167]. These safety data cover a medium-term period and consist of 208,050 patient-years of VDZ exposure [167]. Notably, there were no indications of an overall elevated risk of malignancy in either CD or UC based on these findings [167]. The occurrence of cancer was observed in 140 individuals diagnosed with CD and 123 individuals diagnosed with UC who were undergoing VDZ treatment [167].

Insufficient data are available to determine whether elderly patients with IBD face an increased risk of developing cancer [169]. The Long-term Italian Vedolizumab Effectiveness (LIVE) study examined elderly IBD patients (aged ≥65 years) and clinically matched them in a 1:2 ratio with non-elderly patients (aged 18–64 years), following them for a maximum of 2 years [173]. The study found that elderly patients had a higher likelihood of receiving a cancer diagnosis while undergoing VDZ therapy compared to the non-elderly patients (OR = 4.62; 95% CI, 1.56–12.13; *p* = 0.002). However, this observation could be attributed to the patient’s advancing age rather than the effects of the drug. Additionally, the follow-up period was too short to assess cancer outcomes, similar to other recent studies [173]. Another comparative effectiveness study involving 754 older patients (aged ≥50 years) with IBD from the Danish National Patient Register, consisting of 377 patients using VDZ and 377 using ustekinumab (UST), reported very few cases of new malignant neoplasms (<5 events), making it impossible to compare the safety outcomes between the two drugs [174].

#### 3.2.6. Anti-IL12/IL23

Ustekinumab appears to exhibit a favorable safety profile in patients with IBD that aligns with the findings from available clinical trial data and observational studies as well as recent metanalysis [175,176,177,178,179].

A combined analysis of six phase 2/3 studies conducted on individuals with IBD revealed a rate of malignancies at 0.40 (95% CI, 0.16–0.83) per 100 py, this rate was similar to those observed in the placebo group [175]. Moreover, the IM-UNITI long-term extension study, which followed patients for five years, provided additional evidence supporting the long-term safety of UST: the rate of malignancy was 1.7 vs. 1.06 per 100 py when comparing UST with the placebo, respectively [176].

The interpretation of the results is constrained due to the absence of a suitable reference group, as only 61 patients continued taking the placebo beyond Week 44 in the long-term extension study [176]. For UC, data from a three-year follow-up in the UNIFI program revealed that the overall incidence of malignancy per 100 years of follow-up was 0.72 (95% CI, 0.33–1.36)] for UST and 0.66 (95% CI, 0.08–2.38) for the placebo [177]. However, in both the IM-UNITI and UNIFI program, the small sample size of the placebo reference group during the long-term extension study may impair the validity of these results.

Additional prospective data are necessary to further investigate the higher occurrence of newly developed neoplasms in elderly patients treated with UST, as described in a real-world study using the ENEIDA registry. However, it is important to note that this correlation is likely influenced by the advanced age of the patients involved [178].

#### 3.2.7. JAK Inhibitors

Based on findings from the ORAL Surveillance study comparing tofacitinib to TNFi in rheumatoid arthritis, the FDA included a new ‘black box warning’ on the class of Janus kinase (JAK). ORAL Surveillance was a prospective, phase 3b/4, randomized, open-label, non-inferiority, safety endpoint study comparing tofacitinib and TNFi, which included patients aged at least 50 years old and with at least one additional cardiovascular risk factor [180]. The tofacitinib 5 mg twice daily dose was associated with a numerically higher incidence of all malignancies, excluding NMSC, especially in older patients who smoked [180].

The evidence regarding the risk of malignancy linked to Tofacitinib in patients with IBD is mainly derived from data obtained in the OCTAVE trials (Oral Clinical Trials for tofacitinib in ulcerative colitis) [96,181,182,183,184,185,186]. 

A higher incidence of NMSC was observed in IBD patients who received tofacitinib compared to those who received a placebo [183,184]. In a long-term extension study called OCTAVE Open, which involved 944 IBD patients with a follow-up of up to 7 years, the IRs for confirmed cases of NMSC were as follows: 0.96 (95% CI, 0.35–2.08) for patients on 5 mg tofacitinib, 0.68 (95% CI, 0.35–1.19) for patients on 10 mg tofacitinib, and 0.75 (95% CI, 0.45–1.19) for patients across all tofacitinib doses [184]. Among the types of NMSC, BCC was the most common, with a total of 13 patients primarily belonging to the 10 mg tofacitinib group [183,184].

The Tofacitinib Global Safety Database has reported data from the induction, maintenance, and long-term extension trials, which included a total of 2809.4 py of tofacitinib exposure, up to 7.8 years of treatment, and a median duration of 685.5 days. Overall, NMSC occurred in 21 patients, the most common type was BCC [184].

The recent data from the UC Clinical Program were stratified by age and it was found that the IRs for malignancy in patients aged >65 years (excluding NMSC; IR = 2.05; 95% CI, 0.56–5.25) and NMSC (IR = 3.97; 95% CI, 1.60–8.19) were greater than those for patients aged <65 years (IR = 0.65; 95% CI, 0.37–1.06; and IR = 0.37; 95% CI, 0.17–0.70, respectively) [187]. Prior NMSC and increasing age were significant risk factors for NMSC in the multivariable analysis [187].

A meta-analysis of RCTs did not report differences in the risk of NSMC associated with JAK inhibitors in IBD patients, compared with the placebo or active comparator (RR = 1.21; 95% CI, 0.19–7.65) [188,189].

Filgotinib has been evaluated for use in CD (the FITZROY study) and UC (the SELECTION study) [190,191]. Regarding the safety of filgotinib in IBD, the data available mainly come from clinical trials, particularly the phase IIb/III SELECTION and phase II FITZROY studies [191,192,193,194]. However, these studies did not have sufficient duration to accurately assess its long-term safety, and the results of two phase III studies in CD (DIVERSITY1 and DIVERSITYLTE) will provide evidence in this matter [195,196]. In the MANTA, SELECTION, and SELECTION LTE studies, a total of 20 cases of malignancies and 20 cases of NMSC were observed in patients with UC. The most observed malignancy in UC patients was gastrointestinal neoplasms, while BCC was the most frequent type of NMSC. As already described in tofacitinib-treated patients, the incidence of malignancies other than NMSC was slightly higher in patients aged ≥65 years [194,197,198].

In a series of multicenter phase III trials, two replicate induction studies (U-ACHIEVE induction and U-ACCOMPLISH induction), and a single maintenance study (U-ACHIEVE maintenance), upadacitinib (UPA) proved to be a safe drug in ulcerative colitis treatment, although the authors highlighted the risk of underestimating the risk of malignancy with the restriction to an 8-week induction and 52-week maintenance therapeutic regimen [199]. Recent data from the phase 3 open-label extension study were published comprising 1308 patients, representing 2350.2 py of UPA exposure, and no remarkable differences were observed in the exposure-adjusted incidence rates (EAIRs; n/100 py) of malignancy (excluding NSMC) between the three groups (UPA 15 mg vs UPA 30 mg vs placebo) [200]. More data are needed to confirm the safety profile of upadacitinib therapy in patients with moderately to severely active UC [201].

Recent data from Upadacitinib therapy in CD were published [200]. They included data from the phase 3 clinical program consisting of two induction trials, U-EXCEL and U-EXCEED, and one maintenance trial, U-ENDURE, while the long-term extension trial of U-ENDURE is still ongoing. Similar to the other trials described, the limited exposure association between UPA and the associated risk of malignancy needs to be further investigated in future studies [200].

#### 3.2.8. SP1-Receptor Modulators

The effectiveness and safety of ozanimod in patients with moderately to severely active ulcerative colitis (UC) were shown in both phase 2 TOUCHSTONE and phase 3 True North (TN) studies, which lasted for up to 52 weeks [202,203]. Recently, a partial analysis of the ongoing True North open-label extension (OLE) was published, which included a total of 823 patients corresponding to 2219 patient-years of exposure. The analysis confirmed that malignancies, primarily BCC, were rare occurrences [203]. As previously mentioned, due to the limited observation time and the small sample size of patients analyzed, it is not possible to draw a definite conclusion regarding the connection between ozanimod and specific neoplasms [203].

#### 3.2.9. Dual-Targeted Therapy (DTT)

A systematic review conducted by Ribaldone et al. in 2019 analyzed 18 patients who underwent dual biologic therapy (DBT) [204]. However, due to the limited number of cases, the authors were unable to draw definitive conclusions regarding the safety of DBT [204].

Sands et al. presented the only RCT evaluating the safety of DBT for IBD [205]. In this exploratory multi-center, double-blind, placebo-controlled trial, 52 patients with active CD on IFX had natalizumab added for 10 weeks. The authors found no difference in adverse events [205]. Regarding therapy combining a small molecule with biologic therapy, the phase 2a VEGA study analyzed the safety and effectiveness of combining guselkumab and golimumab for induction therapy compared to using guselkumab or golimumab alone in adults with moderate-to-severe UC [206]. The occurrence of adverse events was similar across all three treatment groups [206].

In 2022, a comprehensive analysis was conducted to investigate this topic, utilizing a systematic review with a meta-analysis approach [207]. The study encompassed a total of 273 patients participating in 279 trials, drawing upon 13 different research studies. However, eight trials involving seven patients were excluded from the analysis because they used biologics or small molecule therapy that had not received approval from the FDA [207]. The results of this analysis indicated that DTT seemed to be a safe and effective therapeutic approach [207]. It is worth noting, however, that the available evidence on this topic remains somewhat limited [207].

A recent systematic review e”amine’ 29 studies that involved 288 patients who were prescribed DTT for the treatment of partially or non-responsive IBD [208]. These studies were divided as follows: fourteen studies included one hundred and thirteen patients who received TNFi and anti-integrin therapies (such as VDZ and natalizumab), twelve studies involved fifty-five patients who received VDZ and UST, nine studies comprised sixty-eight patients receiving VDZ and tofacitinib, five studies encompassed twenty-four patients receiving TNFi and tofacitinib, six studies involved eighteen patients receiving TNFi and UST, and three studies included thirteen patients receiving UST and tofacitinib [208]. However, due to the limited availability of data, no safety assessment could be conducted based on these findings [208].

### 3.3. Therapy Management with Immunomodulators and Biologic Agents in Patients with IBD and Prior or Current History of EICs

Immunomodulators and biologic agents may influence the natural history of malignancies, so the treatment of patients with IBD and prior or current extraintestinal cancer is challenging [13].

There is limited evidence-based data available because most of the clinical trials investigating these drugs exclude patients with active cancer or a recent history of malignancy [13]. Moreover, in studies including patients with IBD and prior or current cancer, patients exposed to IMM or biologics who had prior or current cancer were at lower risk of recurrence or a lower grade than patients not exposed to therapy, so this may represent a selection bias and a difficulty to confront these patients [13].

Indeed, current data regarding the risk of cancer recurrence and the safety profile of therapies mostly derive from studies performed on transplanted patients or patients with other immune-mediated diseases (i.e., rheumatoid arthritis) [13]. We only described studies evaluating patients affected by IBD.

Beaugerie et al. conducted a case-control study to evaluate the impact of IMM on the risk of incident cancer in IBD patients with previous cancer [209]. These patients are at higher risk of developing a new or recurrent cancer when compared to IBD patients without a history of cancer (HR = 1.9; 95% CI, 1.2–3.0; *p* = 0.003), but the exposure to IMM per se is not associated with a significant excess risk of incident cancer [209].

A retrospective study of IBD patients with previous cancer and subsequently treated with IMM and/or biologics demonstrated that exposure to TNFi alone, combination therapy (TNFi plus IMM), or IMM alone was not associated with an increased risk of incident cancer when compared to controls who did not receive IMM (TNFi: HR = 0.32; 95% CI, 0.09–1.09; combination therapy: HR = 0.64; 95% CI, 0.26–1.59; IMM: HR = 1.08; 95% CI, 0.54–2.15) [75].

Holmer et al. conducted a comparison between the safety of TNFi and other classes of biologics or IMM (VDZ, UST, thiopurines, and MTX) in patients with IBD and current or prior cancer [210]. In patients with active cancer, the PFS in patients exposed to TNFi was similar to the PFS of patients exposed to non-TNFα biologics (HR = 0.76; 95% CI, 0.25–2.30; *p* = 0.62) [210]. Again, in patients with prior cancer, there was no difference in the risk of recurrence-free survival (RFS) between patients exposed to TNFi vs non-TNFα biologics (HR = 0.94; 95% CI, 0.24–3.77) [210]. TNFi and non-TNFα biologics exhibit similar safety profiles, with a generally low risk of cancer progression and recurrence [210].

A multicenter cohort study by Poullenot et al. evaluated the risk of incident cancer in IBD patients with previous malignancy and subsequently exposed to TNFi [137]. The incidence of new or recurrent cancer was 84.5 per 1000 patient-years (95% CI, 83.1–85.8). RFS was 96%, 86%, and 66% at 1, 2, and 5 years [137].

In another cohort study of the same scientific group, in IBD patients with prior EICs, incidence rates of new or recurrent cancer were 47.0 per 1000 py for patients receiving no IMM, 36.6 per 1000 py for the TNFi cohort, and 33.6 per 1000 py for the VDZ cohort. RFS did not differ in these three groups [211].

One retrospective cohort study investigating patients with IBD with current or prior malignancy who were subsequently treated with VDZ, TNFi or no biologic reported that neither VDZ nor TNFi was associated with increased risk of incident malignancies when compared to patients not exposed to biologic agents (HR = 0.72; 95% CI, 0.38–1.36, and HR = 1.03; 95% CI, 0.65–1.64, respectively) [212].

Hong et al. performed a retrospective cohort study to compare the risk of incident cancer in patients with IBD and previous cancer treated with UST or VDZ and patients treated with IMM, TNFi, or no therapy [213]. The risk of incident cancer did not differ between patients treated with VDZ and UST (subsequent cancer rate = 1.9 and 3.0 per 100 py, respectively) and patients in the IMM, TNFi, and no therapy groups (subsequent cancer rate =1.6, 3.8, and 2 per 100 py, respectively) [213].

Another retrospective study included patients with IBD and a history of cancer who were subsequently treated with VDZ, UST, TNFi, or no therapy [214]. This study did not show any increased risk of incident cancer in patients receiving treatment with UST (HR = 0.88; 95% CI, 0.25–3.03), VDZ (HR = 0.18; 95% CI, 0.03–1.35) or TNFi (HR = 0.47; 95% CI, 0.20–1.1) [214].

Scott et al. assessed the risk of incident NMSC in patients with IBD and previous NMSC treated with IMM or biologics [159]. Thiopurines and TNFi had a higher risk of incident NMSC when compared to non-exposed (HR = 1.53; 95% CI, 0.87–2.70; and HR = 1.34; 95% CI, 0.64–2.81, respectively). Furthermore, thiopurines appeared to have a higher risk of incident NMSC when compared to the TNFi group (HR = 1.23; 95% CI, 0.78–1.94) [159]. Although, thiopurines used in combination with TNFi did not show an increased risk of incident NMSC when compared to TNFi monotherapy (HR = 0.79; 95% CI, 0.30–2.08) [159].

In a retrospective cohort study by Algaba et al., 133 of 1900 IBD patients developed cancer (*n* = 145) [73]. After cancer diagnosis, patients were maintained on thiopurines, MTX, TNFi, or combined therapy, or discontinued therapy [73]. The rate of death and cancer remission during follow-up did not differ between patients who maintained the treatment compared with patients who withdrew (5% vs 8% and 95% vs 74%, respectively) [73]. An association between thiopurines or anti-TNF-α drugs and cancer was not found [73].

Khan et al. performed a retrospective study on IBD patients who had incident BCC diagnosis and continued or stopped thiopurines or TNFi [215]. Those patients were compared to patients who only took mesalamine: only patients who took thiopurines had a higher risk of new or recurrent BCC (HR = 1.65; 95% CI 1.24–2.19; *p* = 0.0005) [215].

In the meta-analysis by Shelton et al., among 3706 IBD patients, there were 539 cases of incident cancers (including EICs and intestinal cancers). There was no statistical difference (*p* > 0.30) between the IRs of patients on IMM or TNFi or no therapy (37.9 per 1000 py, 48.5 per 1000 py, and 35.7 per 1000 py, respectively) [216].

## 4. Discussion

When compared with the healthy population, patients affected by IBD have a slightly elevated risk of developing solid-organ EICs, including biliary cancers, liver cancer, pancreatic cancer, NMSC, reproductive cancers, urological malignancies, respiratory malignancies, and thyroid cancer [15,15,16,17,18,19,20,22,23,24,26,28,29,30,31,32,37,38,39,40,41,42,43,44,46,47,48,49]. Except for cholangiocarcinoma, there is no evidence to recommend the need for a distinct approach for the screening and surveillance of EICs in patients with IBD, so it is advisable for patients with IBD to adhere to the primary and secondary prevention programs recommended for the general population, based on individual risk [10,14,15,16]. Patients with IBD have also a higher risk of developing hematological malignancies when compared to the general population, but the available evidence is inconclusive in establishing whether this increased risk is attributed to the therapy or other risk factors [10,14,15,16,19,21,28,30,31,34,35,39,40,54,56,57,58,59,60,61].

Evidence suggests that thiopurines may increase the risk of developing lymphoproliferative and myeloproliferative disorders, NMSC, urinary tract cancers, and cervical neoplasia. This risk is influenced by the patient’s specific risk factors, such as older age and smoking, and by the concomitant use of TNFi. However, the overall incidence remains low and recent guidelines conclude that patients treated with thiopurines should adhere to the skin and cervical cancer screening programs recommended for the general population [10,28,70,100,106,107,108,109,121,125,127].

No conclusive evidence suggests a general elevItion in the risk of EICs among IBD patients treated with TNFi [136,138,139,140,148]. However, there may be a higher risk of lymphoma and melanoma [79,100,104,111]. Particularly, combination therapy with TNFi and thiopurines may increase the risk of lymphoma when compared to monotherapy [100,135,139,148,150,154]. Currently, screening measures for the general population are also recommended for these patients and clinicians should encourage them to follow these measures, based on individual risk and clinical judgment [10].

There is no evidence of increased risk of EICs in IBD patients treated with VDZ, UST, or JAK inhibitors, or new small molecules [96,167,168,170,171,171,176,184,203]. Nevertheless, there are scarce long-term data available for patients with IBD, especially for newer drugs such as JAK inhibitors.

Data regarding patients with IBD and prior cancer treated with thiopurines are limited but they do not show a higher risk of recurrence, except for NMSC and BCC [75,159,209,213,215]. Thiopurines may be cautiously initiated, considering the potential risk of cancer recurrence [216]. Patients using thiopurines after cancer should undergo screening examinations following the same guidelines as the general population [10]. Ideally, thiopurines should be discontinued in patients with current cancer, but they may be maintained under vigilant supervision in patients with cancers endoscopically or surgically removed [10,210,215].

The use of TNFi does not seem to be associated with tumor progression, recurrence, or survival outcomes across different malignancies, specifically in melanoma, NMSC, or breast cancer [73,75,137,159,210,211,212,213,215,216]. Nevertheless, it is important to note that the heterogeneity of the study populations, cancer types, and the observational nature of the data limits the application of these results into clinical practice.

VDZ or UST do not appear to increase the risk of incident cancer in patients with IBD and prior cancer, but limited data are available [210,211,212,213,214]. Currently, the available evidence is inadequate to establish recommendations regarding the utilization of JAK inhibitors in patients with prior cancer [10,96]. Insufficient data are also present concerning the safety of VDZ, UST, or JAK inhibitors in individuals with current malignancies [10].

The issue of using different effect measures in the studies considered in this review is an important aspect to consider when interpreting and comparing the findings [217]. The choice of effect measure often depends on the study design, the available data, and the specific research question being addressed [217]. However, this variability in effect measures can introduce challenges in synthesizing and comparing results across different studies [217].

## 5. Conclusions

Patients with IBD have a higher risk of developing different forms of EICs and hematological malignancies. Immunomodulators and biological therapy may increase the risk of developing some types of EICs and may be consciously used in patients with IBD and current or prior history of malignancy. The treatment of patients with IBD should be based on a risk-benefit assessment, based on the risk profile of the specific drug, disease severity, as well as patient characteristics, considering the potential factors that may increase the risk of neoplasia. Regrettably, the lack of adequate information on each drug often hinders the ability to make a well-informed decision, adding to the difficulty of the process. Decisions regarding the use of immunomodulators or biological therapies in patients with a history of EICs should be made on an individual basis, considering a multidisciplinary approach involving oncologists. Factors such as the patient’s current and recent IBD activity, as well as alternative treatment options, should be carefully considered. Further studies are needed to obtain more evidence regarding the association between immunomodulators and biological therapies and the risk of developing malignancies to help specialists with clinical practice.

## Figures and Tables

**Table 1:** Studies investigating the association between extraintestinal solid-organ tumors and IBD.

Study	Year	Country	Study Design	Main Findings
Ekbom et al. [17]	1991	Sweden	Population-based study	CD: increased risk of squamous skin cancer (SIR 5.5). UC: increased risk of connective tissue (SIR 4) and brain (SIR 2.4) cancers.
Persson et al. [18]	1994	Sweden	Population-based study	Increased risk of bladder, lung, brain, and skin cancers.
Mellemkjaer et al. [19]	1995	Denmark	Population-based study	Increased risk of NMSC (RR 1.4) and hepatobiliary cancers (RR 2.3).
Bernstein et al. [20]	2001	Canada	Population-based study	Increased risk of liver and biliary tract cancers (IRR 3.96–5.22).
Jess et al. [21]	2004	Denmark	Population-based study	No increased risk of EICs.
Bhatia et al. [22]	2006	USA	Retrospective study	Correlation between IBD and cervical abnormalities.
Hemminki et al. [23]	2008	Sweden	Population-based study	UC: increased risk of liver (SIR 4.30), breast (SIR 1.25), and prostate (SIR 1.14) cancers.
Hemminki et al. [24]	2009	Sweden	Population-based study	CD: increased risk of liver, testis, and kidney cancers (SIR > 2).
Singh et al. [25]	2009	Canada	Population-based study	No association between IBD and cervical abnormalities.
Erichsen et al. [26]	2009	Denmark	Cohort study	Increased risk of cholangiocarcinoma (four-fold).
Lees et al. [27]	2009	UK	Case-control study	No increased risk of cervical abnormalities.
Long et al. [28]	2012	USA	Retrospective study	Increased risk of melanoma (IRR 1.29) and NMSC (IR 1.46).
Jussila et al. [29]	2013	Finland	Population-based study	CD: increased risk of biliary tract cancers (SIR 4.93). UC: increased risk of thyroid (SIR 1.93) and biliary tract (SIR 7.23) cancers. IBD: increased risk of basal cell skin cancers (SIR 1.29).
Jess et al. [30]	2013	Denmark	Population-based study	CD: increased risk of lung cancer (SIR 2.13) and cervical dysplasia (SIR 1.65). UC: increased risk of prostate cancer (SIR 1.82).
Kappelman et al. [31]	2014	Denmark	Population-based study	IBD: increased risk of smoking-related cancers (SIR 1.5), melanoma (SIR 1.4), and NMSC (SIR 1.8). CD: increased risk of gallbladder and biliary tract (SIR 2.4) cancers. UC: increased risk of liver (SIR 1.6) and gallbladder (SIR 2.5) cancers.
Rungoe et al. [32]	2015	Denmark	Population-based study	Increased risk of SILs and cervical cancers (IRR 1.15–1.55).
Kim et al. [33]	2015	USA	Population-based study	No association between IBD and cervical abnormalities.
van den Heuvel et al. [34]	2016	The Netherlands	Population-based study	CD: increased risk of skin cancer (SIR 1.55); decreased risk of breast cancer (SIR 0.11). UC: no increased risk of EICs.
Wilson et al. [35]	2016	UK	Population-based study	No increased risk of EICs.
Madanchi et al. [36]	2016	Switzerland	Retrospective study	Increased risk of urothelial cancer and cholangiocarcinoma.
Wadhwa et al. [37]	2016	USA	Case-control study	Increased risk of thyroid cancer (OR 1.97).
Hovde et al. [38]	2017	Norway	Population-based study	CD: increased risk of trachea/lungs cancer (SIR 2.91). UC: increased risk of breast (SIR 2) and liver/biliary (SIR 2.85) cancer; reduced risk of lung cancer (SIR = 0.79).
So et al. [39]	2017	China	Population-based study	CD: increased risk of renal-cell carcinoma (SIR 6.89), head and neck and CNS cancer (SIR 5.08), and NMSC (SIR 13.88). UC: increased risk of prostate cancer (SIR 2.47) and NMSC (SIR 9.05).
Jung et al. [40]	2017	Republic of Korea	Population-based study	CD: increased risk of liver (SIR 15.3) and pancreatic (SIR 8.6) cancers for women. UC: increased risk of prostate (SIR 3.5), CNS (SIR 6.1), and thyroid (SIR 2.2) for men; liver (SIR 4.4) and cervix uteri (SIR 5.7) for women.
Mosher et al. [41]	2018	USA	Case-control study	Increased risk of NMSC (RR 2.38), melanoma skin (RR 2.85), renal (RR 2.9), prostate (RR 1.7), and pancreatic (RR 4.23) cancers.
Loo et al. [42]	2019	Canada	Population-based study	Increased risk of breast (SIR 1.13), respiratory (SIR 1.16) cancers, and NMSC (SIR 22.62).
Burns et al. [43]	2019	USA	Retrospective study	Increased risk of prostate cancers (HR 4.84).
Taborelli et al. [44]	2020	Italy	Population-based study	UC: increased risk of corpus uteri (SIR 2.67) and kidney (SIR 2.06) cancers. CD: increased risk of thyroid cancer (SIR 5.58) and NMSC (SIR 1.86).
Everhov et al. [45]	2020	Denmark, Sweden	Population-based study	Increased risk of pancreatic cancer (SIR 9.04).
Wang et al. [46]	2021	China	Cohort study	Thyroid, cervical, hepatobiliary, and urinary tract cancers were the most common EICs. Patients with elderly-onset IBD are at higher risk of EICs (RR 2.83).
Goetgebuer et al. [47]	2021	The Netherlands	Case-control study	Increased risk of CIN2+ (SDR 1.27).
Wang et al. [48]	2022	United States	Population-based study	IBD: increased risk of lung cancer (OR 1.14) and melanoma (OR 1.19).

Abbreviations: CD Crohn’s disease; CIN, cervical intraepithelial neoplasia; CNS, central nervous system; EICs, extraintestinal cancers; HR, hazard ratio; IR, incident rate; IRR, incidence rate ratio; IBD, inflammatory bowel disease; NMSC, non-melanoma skin cancer; OR, odds ratio; RR, risk ratio; SIR, standardized incidence ratio; UC ulcerative colitis.

## Data Availability

The data presented in this study are available in this article.
